# Ten Open Questions about Laser-Induced Periodic Surface Structures

**DOI:** 10.3390/nano11123326

**Published:** 2021-12-07

**Authors:** Jörn Bonse, Stephan Gräf

**Affiliations:** 1 Bundesanstalt für Materialforschung und -prüfung (BAM), Unter den Eichen 87, D-12205 Berlin, Germany; 2 Otto-Schott-Institut für Materialforschung (OSIM), Löbdergraben 32, D-07743 Jena, Germany

**Keywords:** laser-induced periodic surface structures, industrial application, open questions, modelling, functional properties

## Abstract

Laser-induced periodic surface structures (LIPSS) are a simple and robust route for the nanostructuring of solids that can create various surface functionalities featuring applications in optics, medicine, tribology, energy technologies, etc. While the current laser technologies already allow surface processing rates at the level of m^2^/min, industrial applications of LIPSS are sometimes hampered by the complex interplay between the nanoscale surface topography and the specific surface chemistry, as well as by limitations in controlling the processing of LIPSS and in the long-term stability of the created surface functions. This Perspective article aims to identify some open questions about LIPSS, discusses the pending technological limitations, and sketches the current state of theoretical modelling. Hereby, we intend to stimulate further research and developments in the field of LIPSS for overcoming these limitations and for supporting the transfer of the LIPSS technology into industry.

## 1. Introduction

Inspired by the historic idea of David Hilbert to formulate and collect the most relevant open problems in his field [1], we aim to provide here a list of currently open questions related to the research and technology of laser-induced periodic surface structures (LIPSS, sometimes referred to as ripples). LIPSS manifest as grating-like surface morphologies typically exhibiting sub-micrometric spatial periods *Λ* (either imprinted in the surface topography, or in the structural or chemical state of the near-surface material). They are a universal phenomenon occurring during the processing of solids by intense laser radiation [2]. While initially seen as an unexpected effect during the early developments of lasers [3], and later rather as a side-effect reducing the precision of materials processing [4], the LIPSS phenomenon is currently considered a simple nanostructuring approach that—if properly controlled—may enable a plethora of promising technical, biological, or medical applications via tailored surface functionalization [5,6,7]. The LIPSS technology currently stays at the threshold of entering industrial applications. For that, however, some immanent problems should be overcome.

This list of ten open questions must be a subjective compilation of the authors and, thus, this article is formulated as a “Perspective.” With this paper, we complement and extend our previous review articles [5,6,7,8,9,10,11] and hope to contribute to the identification of relevant open and partly controversially discussed questions in the field of LIPSS, while simultaneously stimulating further research activities in our community that are helping to resolve barriers, which currently prevent the LIPSS technology from widely entering into industrial applications.

## 2. Ten Open Questions about LIPSS

**Question** **1.**  **Can LIPSS be Generated on Every Material?**

**Figure 1:**
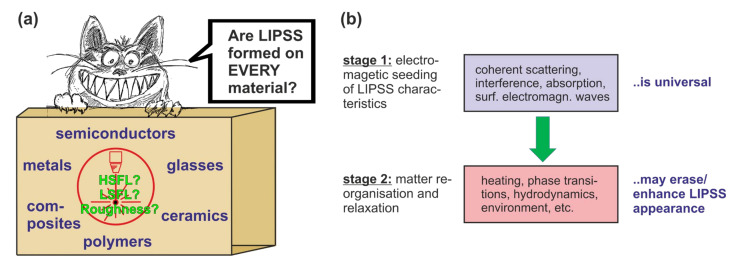
(**a**) Humoresque sketch of the question of whether LIPSS can be formed on every material? (**b**) Abstraction of the LIPSS formation in two subsequent stages (electromagnetic seeding vs. matter re-organisation) affecting the final LIPSS appearance.

Probably one of the most discussed topics within the LIPSS community is the question whether LIPSS can actually be produced or generated on all materials (see Figure 1a)? The question is not only about the feasibility of generating the periodic laser-induced structures. Rather, a key aspect is given by the option of defined control of the structure’s properties, especially with regard to industrial and commercial applications. This includes the spatial period, the defined alignment, and the modulation depth of the nanostructures. In order to address the topic, it is first necessary to classify the different types of LIPSS, which has already been undertaken in detail in numerous review articles [5,8,9,10]. Briefly, the basic classification involves LIPSS with low-spatial frequency (LSFL) and high-spatial frequency (HSFL), where the period *Λ* of LSFL is of the order of the utilized laser wavelength (*Λ* ~ *λ*) and for HSFL much smaller structures with *Λ* << *λ* are achievable. Besides LSFL and HSFL also supra-wavelength sized “grooves” and “spikes” are reported in the pertinent literature of self-organized laser-generated surface structures [12,13].

A review of experimental work over the last decades leads to the conclusion that LSFL can be produced on all types of material including metals, semiconductors, dielectrics (glasses, ceramics, polymers) and also composite materials, thin films, or 2D nanomaterials [14,15,16,17,18,19,20]. In this context, numerous studies have investigated LIPSS formation in single-spot experiments, in which a fixed location on the material surface is exposed to a specific number of laser pulses without relative movement. The evaluation of these basic investigations is usually carried out as a function of the typical influencing parameters such as the polarization state [21,22,23,24], laser wavelength [25], number of pulses [26], pulse repetition frequency [27], laser peak fluence [9], and the ambient medium [28,29,30]. Driven by potential industrial applications, large-area surface structuring is moving more into the focus of investigations. Experimental studies can be found here mainly for metals due to the linear absorption behavior [31]. Recent progress has also been made for materials showing nonlinear absorption (e.g., glasses) including the application of absorption-mediating metallic thin films [32]. Since HSFL have so far mainly been demonstrated on large-band-gap materials upon irradiation with ultrashort laser pulses and on several metals [14], the suitable range of materials is a priori limited here.

However, the experimental work proves that the optical properties and the electronic structure of the materials play a central role in answering the initial question. The optical constants are also the basic input parameters of the electromagnetic models (see Question 10), which from a theoretical point of view describe the LIPSS formation based on the scattering and interference at the rough material surface. In this context, the rigorous model provided by Sipe et al. in 1983 [33] is still the most widely accepted analytic approach. Supported by modern computing technology, it has been extended in recent years by Finite-Difference Time-Domain (FDTD)-based numerical calculations (see Question 10) [34,35,36]. Taking into account the specific optical constants (dielectric permittivity *ε*) as well as near- and far-field scattering effects, these electromagnetic approaches predict that LIPSS formation should, in principle, be possible on all materials. The experimental studies, however, reveal that the capacity of a material to form well-pronounced LIPSS can vary greatly even on materials within the same material class. For metals, Gnilitskyi et al. [31] showed that the regularity of the structures depends remarkably on the optical properties that also vary with the laser irradiation wavelength (see Question 4). Glasses and polymers are characterized by their strongly nonlinear interaction of the material excitation for laser wavelengths in the visible and infrared spectral ranges and through material-specific incubation effects. Therefore, the formation of LIPSS, especially on large surface areas, remains a challenging problem. This is related to the significant impact of other material parameters such as the internal structure (amorphous/crystalline) and other thermophysical parameters affecting hydrodynamic motion at the laser-melted surface. The latter can significantly influence the behavior of the material during LIPSS formation, e.g., through temperature-dependent viscosity or surface tension changes and the decomposition of the material. Thus, for example, small-scale HSFL are seldom observed on temperature sensitive polymeric materials [37,38]. To answer the initially raised question, as a general rule one can state that the electromagnetic scattering and absorption effects (stage 1 in Figure 1b) are a universal mechanism seeding the characteristics of the LIPSS [2,35,36,39], but the subsequent material-specific re-organization and relaxation effects (stage 2 in Figure 1b) may later prevent (or sometimes even promote) their experimental observation.

Consequently, despite the availability of powerful model approaches and computing technology (see Question 10), in praxis the investigation of LIPSS formation on new materials remains usually an iterative testing and optimization process. Even for materials that have already been studied in detail, one still encounters unresolved, open questions due to the complexity of the processes involved.

**Question** **2.**  **What is the Minimum Structural Size of LIPSS?**

Many types of LIPSS show a characteristic scaling of their spatial period *Λ* with the irradiation wavelength *λ*. Most prominently, both types of LSFL (type I and II [9,10]) scale linearly with *λ* for wavelengths ranging from the vacuum ultraviolet to the far infrared spectral region [7,10]. In contrast, the HSFL exhibit a weak dependence on the irradiation wavelength only. Spatial periods of less than 100 nm were reported [40,41]. From the “electromagnetic point of view,” for strong absorbing materials, such as metals, some lower limit of the spatial period should be imposed by the attenuation lengths of the radiation intensities that account for a few tens of nanometers in the vertical direction (skin depth) and for similar values for the lateral/radial decay of the optical near-fields around optically scattering surface defects [42].

**Figure 2:**
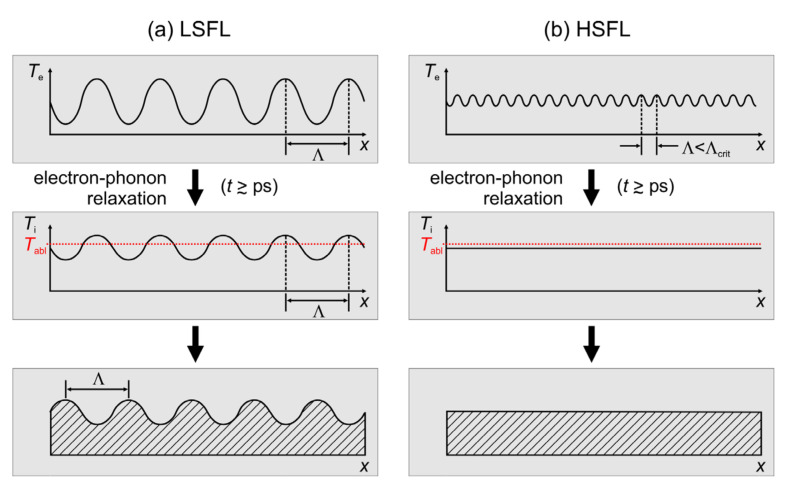
(**a**) Transfer of the LSFL-sized spatial modulation (period *Λ*; imprinted by optical effects) in the electron temperature field (*T*_e_, top) via electron–phonon relaxation to the lattice temperature field (*T*_i_, middle), finally leading to an LSFL surface relief via spatially modulated ablation (bottom). (**b**) Small-scale HSFL-sized spatial modulations of the electron temperature are washed out during electron–phonon relaxation if the spatial period is below a critical threshold value *Λ*_crit_.

Moreover, for ultrashort laser pulse durations, another limiting aspect is caused by the electron–phonon relaxation process that is initiated by the laser-irradiation [9,10]. For very small surface structures, such as HSFL, thermal diffusion effects can wash out the initial spatial modulations in the absorbed energy profile already during the material-specific time of transferring the energy from the optically excited electronic system to the lattice of the solid, i.e., during the electron–phonon relaxation time *τ*_e-ph_ (Figure 2).

However, numerical calculations based on the two-temperature model (TTM) confirmed already for semiconductors and metals and at laser fluences including the melting regime that the spatial LSFL-I characteristics featuring typical periods of several hundred nanometers are preserved during the electron–phonon coupling [43,44].

Nevertheless, detailed studies elucidating the situation for HSFL-sized spatial modulations of the absorbed energy pattern via a TTM model for confirming/refuting the impact of *τ*_e-ph_ on the minimal period limit of HSFL spatial periods are still missing.

**Question** **3.**  **How can the Regularity of LIPSS be Uniquely Quantified?**

Although usually not quantified, the regularity is an essential property of LIPSS as it plays a central role for numerous applications, especially in the field of optics. On one hand, it is determined by the regularity of LIPSS generated within the single focal spot of the laser processing beam. On the other hand, it also depends significantly on the coherent linking of the LIPSS during large-area structuring, when laser beam scanning strategies are applied [45]. Both from the point of view of theoretical modelling, which attempts to describe in detail the interaction of the laser and process parameters with the optical, structural, and topographical properties of the initial surface, and from the point of view of potential applications, an objective and reliable analysis of the characteristic LIPSS properties and the regularity is essentially lacking.

**Figure 3:**
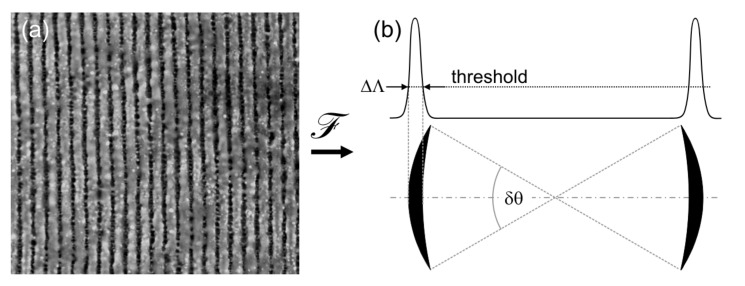
The characterization of LIPSS-covered surfaces is based on SEM/AFM images (**a**) that are used to calculate (*ℱ*) the Fourier spectrum (**b**) from which certain quantities are specified.

The most widely established procedure of LIPSS characterization is based on the evaluation of scanning electron microscopy (SEM) and atomic force microscopy (AFM) images obtained from the structured material surface (Figure 3a). As the most important step, this method includes the calculation of the Fourier spectra using two-dimensional (2D) Fourier transformation (Figure 3b). These Fourier spectra represent 2D-histograms of the spatial frequencies (*k*_x_, *k*_y_ ~1/*Λ*_x,y_) present in the original images. They contain specific features (peaks, schematically represented by the two sickle-shaped black arcs in the lower part of Figure 3b) on which the evaluator individually specifies certain quantities: The center position (*k*_x_, *k*_y_) of the features determines the most frequent spatial periods *Λ* and the LIPSS orientation relative to the laser beam polarization. The opening angle of the features *δθ* is a measure of the angular deviation from the ideally ordered LIPSS grating pattern with *δθ* ~ 0. Moreover, the width of the features Δ*Λ* represents the “dispersion” of the spatial frequencies (periods) that can be observed on the entire imaged surface pattern. Its quantitative value, however, depends on the threshold that is subjectively set. Consequently, the Fourier spectrum corresponds to a characteristic fingerprint of the laser structured material surface, which enables the clear identification of the corresponding LIPSS type along with its geometrical characteristics.

The described procedure for evaluating the opening angles *δθ* of the sickle-shaped LIPSS features in the Fourier space for qualifying the regularity of LIPSS was first introduced by Bonse et al. [46] in their studies on InP. Later, Gnilitskyi et al. [31] performed theoretical model calculations where they used *δθ*, named as dispersion of LIPSS orientation angle (DLOA), as a characteristic parameter to correlate the LIPSS regularity on different metals with their intrinsic optical properties (dielectric permittivity *ε*). He et al. [47] explored in more detail typical morphological defects limiting the regularity of LSFL via bifurcations, bending, and splitting. The authors provided a classification of defects and established a relation to the laser fluence used for laser processing.

Meanwhile, *δθ*/DLOA has developed into a frequently used, practical analysis tool for LIPSS patterns. For LSFL (types I and II), the procedure usually gives reliable values at least within a certain scientific study or working group. Moreover, particularly for HSFL-II in praxis very “broad and noisy” features are obtained in the Fourier space, which makes their quantitative evaluation rather challenging and difficult.

Nevertheless, the main problem of the inevitable individual influencing variables (such as the threshold for quantifying *δθ*) remains, which makes it difficult to compare the results of theoretical and experimental studies and among different groups, respectively. It would be desirable to have a method for calculating reproducible parameters of the LIPSS properties, especially the regularity of arbitrary laser-induced surface structures, independently of the evaluator. Although a first step in this direction was made recently by Lechthaler et al. [48] in the field of Direct Laser Interference Patterning (DLIP), where the authors demonstrated a systematic quantitative analysis of the regularity based on the so-called Gini coefficient for various structures, this is currently not available in the field of LIPSS.

**Question** **4.**  **How can the Regularity of LIPSS be Controlled?**

Unique parameters quantifying the regularity of LIPSS are not established in the field of LIPSS (see Question 3). Thus, the surface morphology is typically optimized in a trial-and-error approach via the iterative variation of experimental processing parameters until an operator (person) decides to accept the quality of a certain LIPSS pattern. The most relevant parameters are (i) laser parameters, such as the spatial beam profile, its peak fluence *F*_0_, the pulse repetition frequency ν, but also (ii) beam scanning parameters, such as the spatial pulse (spot) overlap Δ*x* that determines the effective number of laser pulses *N*_eff_ = 2*w*/Δ*x* per focused beam spot diameter 2*w*, the line separation Δ*y* between adjacent scan lines, the direction of the linear laser beam polarization relative to scanning direction *x* [49], etc.

**Figure 4:**
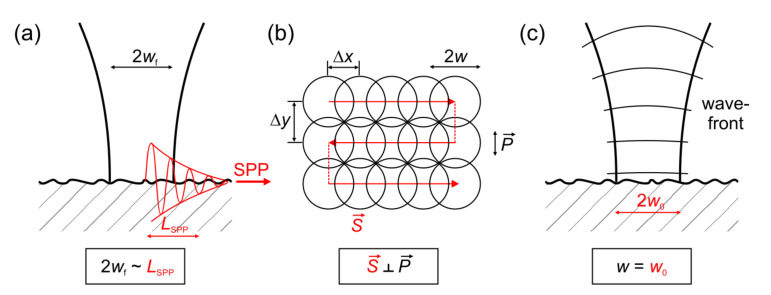
Scheme of how the regularity of LIPSS (LSFL-I) can be improved: (**a**) Adjust the focus diameter 2*w*_f_ to the SPP propagation length *L*_SPP_; (**b**) Adjust the scan direction ***S*** perpendicular to the direction of linear polarization ***P***; (**c**) Chose a flat wavefront by working in the focus.

However, for the LSFL-I, which often rely on a plasmonic (surface plasmon polariton (SPP)-based) formation mechanism [35,39], the latter imposes specific constraints and an intrinsic dependence of the LIPSS regularity on the irradiated material and its specific optical properties (dielectric permittivity *ε*) that is given by the electronic band structure of the solid. These influences of the optical properties on the regularity of LSFL-I were explored theoretically and experimentally by Gnilitskyi et al. for a set of different metals [31]. The authors underlined that for obtaining large-area regular LSFL-I patterns it is key to adjust the laser spot size 2*w*_f_ used for processing to values close to the characteristic material-specific SPP decay length *L*_SPP_ (Figure 4a). This is another elaborated aspect of the early observation of Fauchet and Siegman, who reported already in 1982 that, upon scanning a focused ps-laser beam across the surface of the semiconductor GaAs, for overlapping laser spots (Δ*x* < 2*w_f_*) in a scanned line, the resulting LIPSS ridges can be coherently linked together to form an extended LIPSS pattern [45].

For the same reason, i.e., the excitation of plasmonic surface electromagnetic waves, Ruiz de la Cruz et al. observed for fs-laser processed Cr-films a strong dependence of LSFL-I regularity on the direction of the linear laser polarization (***P***) with respect to the scan direction (***S***): since the scattering of SPP at surface defects exhibits specific directional characteristics, LIPSS processed with ***S*** perpendicular to ***P*** can be significantly more regular than in the case that ***S*** is parallel to ***P*** [49]; see (Figure 4b). In view of these directional SPP scattering characteristics, it becomes also clear that—apart from the scanning direction ***S*** relative to ***P***—the line separation Δ*y* must be also carefully adjusted for optimizing the LIPSS regularity.

San-Blas et al. demonstrated experimentally that the (de-)focusing of a pulsed fs-laser beam by a cylindrical lens has an impact on the regularity of the LIPSS formed on steel surfaces [50]. The authors observed the best regularity for plane wavefronts of the focused laser beam, i.e., either in the focal position (Figure 4c) or when working far away from it. This finding again pinpoints the relevance of coherence in optical scattering and the excitation of SPP during the generation of LSFL-I. Thus, although the control of coherent scattering effects is always the key to obtaining LIPSS of high regularity, the implementation of a general algorithm for controlling the regularity of LIPSS is still missing. Here, artificial intelligence and machine learning approaches [51] could contribute to improving LIPSS regularity in the future.

Finally, it should be noted that the regularity of non-ablative LIPSS, e.g., of LSFL-I originating from localized laser-induced melting and rapid solidification in an amorphous material state [52], or of LSFL-II originating from laser-induced surface oxidation [53,54,55] usually feature much better LSFL regularity than ablative LSFL. For non-ablative LIPSS, this can be explained by their surface characteristics that do not significantly generate optical scattering or diffraction at a somewhat irregular grating-like surface topography (as in the case of ablative LIPSS) but at a rather smooth spatial surface modulation in the form of a superficial refractive index grating. Thus, the type of LIPSS (ablative vs. non-ablative) imposes some additional constraints that intrinsically affect their regularity.

**Question** **5.**  **What Creates the Functionality of LIPSS-Structured Surfaces: Topography vs. Chemistry?**

One of the main difficulties of practical applications of LIPSS arises from the fact that the desired surface functionality is usually affected not only by the grating-like surface topography but also by its specific surface chemistry. The latter includes laser-induced chemical alterations, e.g., superficial oxidation [16,55,56,57] caused by laser-processing in an air environment but also from redeposited material (debris) [58] or from post-irradiation adsorption of molecules such as hydrocarbons during sample handling and ambient storage [58,59,60,61]. Thus, there has been a long-lasting debate in the LIPSS-community regarding the relevance of the topography vs. the interfacial chemistry for surface functionalization. It must be noted, however, that certain types of LIPSS (e.g., LSFL-II on oxidation-prone materials) do in fact originate from the formation of a superficial oxide layer [30,53,54,55,62], while other types of LIPSS (e.g., LSFL-I or HSFL-II on metals) do not specifically rely on surface oxidation effects but may be accompanied by it upon laser-processing in an air environment [16,56,63,64].

**Figure 5:**
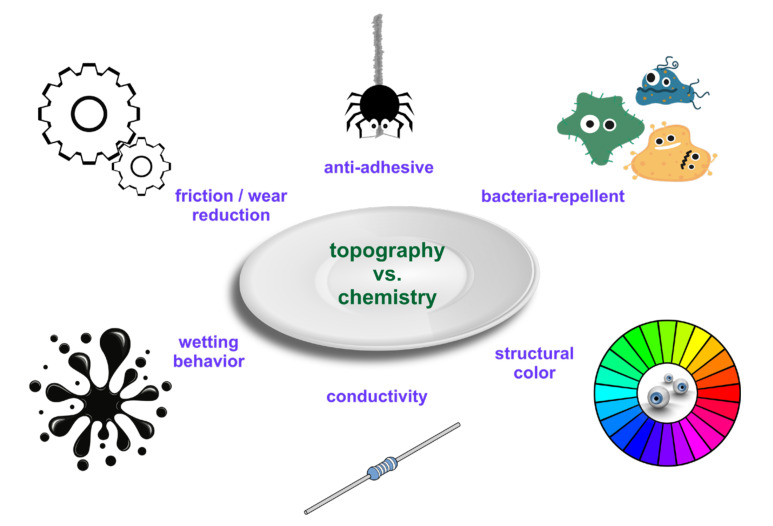
Topography vs. chemistry—a schematic pointing to effects and properties affecting applications of LIPSS.

Figure 5 visualizes several applications of LIPSS, which are affected by both the surface topography and the surface chemistry. One of the first and most obvious applications of LIPSS arises from their ability to diffract light of the visible spectral range [65]—an effect referred to as “structural color.” This optical diffraction, however, imposes a specific angular dependence of the colorized light, which is distinctively different from the usual “interference-based colors” of thin transparent oxide layers or even the response of scattering nanoparticles [65].

Another branch of promising applications of LIPSS is related to their ability to suppress the formation of biofilms [66,67,68,69]. This bacteria-repellence strongly depends on the type of bacteria and specific growth conditions and needs further exploration, particularly for distinguishing between the influence of laser-induced topographic and chemical surface alterations.

Many applications of LIPSS are “inspired by nature” [70] since evolution has developed many surface specific functionalities through tailored rippled surface topographies—often in combination with a specific organic surface chemistry. A less known example is related to the reduction of surface adhesion affects. So-called cribellate spiders exhibit a specific “tool,” the *calamistrum*, at some of their legs to handle the silk [71]. This silk consists of very thin (a few tens of nanometers) nanofibers that, thus, feature strong van der Waals forces to catch the prey. The spider’s calamistrum minimizes the contact area and reduces the adhesive van der Waals forces [71]. Through a European research project (“BioCombs4Nanofibers” [72]) this concept is currently being explored to transfer it to technical surfaces. Joel et al. mimicked this feature by processing LSFL-II on PET foils, rendering these foils anti-adhesive for spider silk [71].

LIPSS can exhibit a beneficial tribological performance by reducing friction and wear. For a recent overview article related to laser-textured metals the reader is referred to [73]. The interplay of LIPSS with tribological effects is manyfold. Laser processing can (i) modify surface roughness [15], (ii) cause near-surface structural changes such as amorphization or re-crystallization that affect, for example, material hardness, (iii) create superficial oxidation layers that may prevent the direct contact of two bodies in relative motion [74], (iv) can alter the surface wetting behavior for lubricants [16], (v) or could even confine some lubricant in the tribological contact area. From the three latter points it becomes clear that particular attention must be paid to the specific surface chemistry and the resulting wetting behavior of LIPSS.

The wetting behavior of various types of laser-textured surfaces, including LIPSS, is currently very actively studied by many researchers. It has become clear already that, e.g., for metals, surface oxidation often causes hydrophilic surfaces immediately after the laser-irradiation, which then—through the adsorption of molecules from the local storage environment—turn into hydrophobic surfaces on the timescale of several days to several weeks after the treatment [58,59]. Hence, it is very difficult to stabilize the surface wetting state—a prerequisite for industrial applications—see Question 6, which is particularly devoted to this eminently practical aspect.

Another aspect that has very recently come to the attention of research on LIPSS is related to the electric and magnetic properties induced by LIPSS [63,75,76,77]. For example, Lopez-Santos et al. demonstrated a directional dependence of the electric resistivity with respect to the orientation of LSFL-I ridges on processed thin indium-doped tin oxide (ITO) films used in photovoltaics [76]. Cubero et al. evidenced that the superconductive properties of niobium sheets (tapes) can be affected by LSFL-I [63,78]. However, compared to the other applications discussed here, so far little research has been conducted in that direction. It deserves, however, a deeper exploration, particularly in view of future energy saving applications or for sensors [79].

**Question** **6.**  **How Can the Wettability of LIPSS-Covered Surfaces be Stabilized in the Long Term to be Reliable in Everyday Life?**

Numerous animals and plants in nature, such as the springtail or the lotus leaf, make use of sophisticated solutions to achieve certain wetting phenomena. In order to transfer nature’s fundamental principles to technical applications, an approach of the current research is based on LIPSS, as these are well suited to realize certain wetting states on different material surfaces [8,70]. LIPSS allow the realization of a wide range from almost complete wettability (super-hydrophilic behavior with a water contact angle *CA* ~ 0°), over hydrophobic behavior, to superhydrophobic, strongly water-repellent surface properties featuring *CAs* > 150°. Polished metals, e.g., are characterized by a slightly hydrophilic behavior. According to Wenzel’s law it is increased by laser structuring due to an increase in surface roughness [80]. However, previous work shows that the achieved contact angles cannot be described solely by topographical aspects, which is why the influence of surface chemistry has to be considered (see Question 5). During LIPSS generation in air environment, metals are subject to laser-induced oxide-layer formation, which favors the formation of a hydroxylated layer and is thus responsible for super-hydrophilic properties after laser processing [81].

Independent of the physical and chemical aspects, the surface wetting analysis is also subject to systematic and subjective influencing parameters. These include, among others, the utilized test method, the applied liquid, its drop volume, and the elapsed time between drop placing and measurement [82,83]. An additional significant influence results from the sample handling, as there is no commonly accepted cleaning procedure with regard to time (before and after structuring, before wetting measurement, no cleaning) and cleaning agent (isopropanol, ethanol, acetone) [84]. These points contribute significantly to the fact that studies from different groups are difficult to compare with each other and that some wetting phenomena are still controversially discussed despite the numerous studies available. A prominent example of this is the transient change in the wetting of metal surfaces after laser structuring, which was first described by Kietzig et al. [59]. Within a few days to weeks this process, known as the “ageing effect,” results in a transition from the initial hydrophilic state to hydrophobic wetting behavior with contact angles of up to 140°. In the literature, the ageing is attributed to the hydrophilic, reactive character of the laser-structured surface, which leads to the formation of a new chemical layer with altered wettability through the adsorption of organic species and the accumulation of hydrocarbons from the surrounding atmosphere [59,85].

Even if the described processes could be considered in the technological manufacturing processes, the main problems of the long duration of aging, poor controllability, and reproducibility, as well as the dependence on the composition of the surrounding atmosphere are still existent. Moreover, Gregorčič et al. found that the final wetting state of laser-structured surfaces achieved by ageing is stable only at first glance [86]. For longer observation times, the hydrophobic state on stainless steel remained stable in the long-term only above a certain threshold fluence, while for laser fluences below this threshold a renewed decrease after reaching the maximum contact angle was reported. This clarifies that the stability of the wetting depends not only on the material but also on the laser and process parameters used.

**Figure 6:**
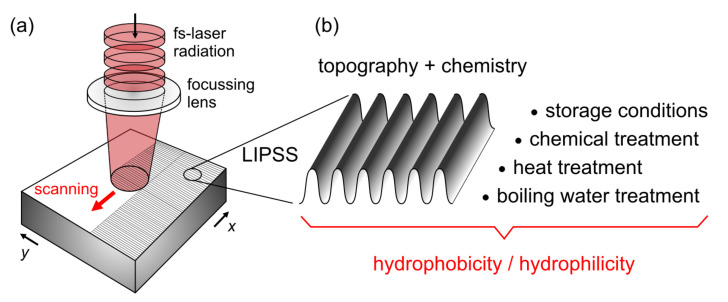
(**a**) Scheme of LIPSS processing in a beam-scanning approach and (**b**) factors affecting and controlling the wettability (e.g., hydrophobicity/-philicity) and long-term stability of LIPSS-structured surfaces.

An interesting question is, therefore, which methods are suitable to adjust the wetting state during/after laser structuring and how to achieve a long-term stability (Figure 6). First approaches use different gases (e.g., air, Ar, CO_2_, vacuum) during laser irradiation [58,87], the subsequent storage of the samples within specific atmospheres [58,59], the immersion of the structured surfaces in boiling water [58,59,88], and heat treatment at different temperatures [89,90]. In addition, the deposition of specific molecular monolayers that covalently bond to the structured surface has become established as a chemical surface modification [89,91]. For this purpose, silanes are mainly used currently, which, depending on the choice of functional groups, reduce or increase the surface energy and, thus, allow the realization of both hydrophobic and hydrophilic wetting states. Despite these promising methods, in everyday life the point of long-term stability of the wetting state usually remains unsolved, as the surfaces are exposed to harsh environmental conditions in industrial and commercial applications. For coatings in particular, abrasion as well as chemical and thermal influences are limiting factors [92].

**Question** **7.**  **Can LIPSS Properties be Modulated/Switched Dynamically?**

For many real-life applications, the dynamic control of the state of a technical system is required. This may include continuous changes of its properties but can also be based on a switching between discrete system states (e.g., as of paramount importance in electronics). This raises the question whether and how properties that are generated by LIPSS can be controlled, dynamically modulated, or even switched by outer parameters. Although many studies on LIPSS are already available, this question is still widely unexplored. For affecting properties resulting from LIPSS, electric or magnetic fields are most desirable due to the compatibility with existing technologies, i.e., microelectronics or photonics.

**Figure 7:**
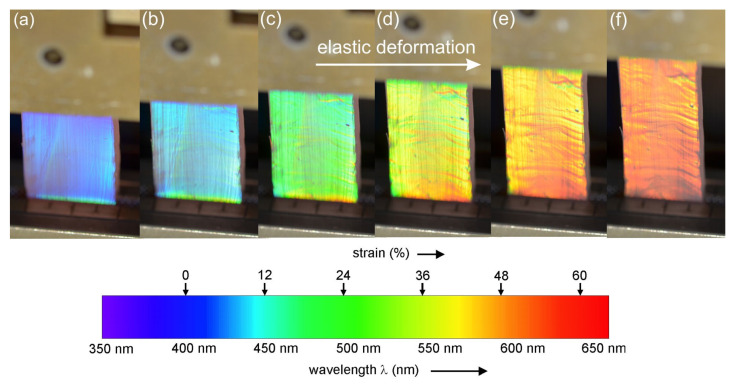
Collage of photographs visualizing structural colors resulting from LIPSS imprinted on polyvinyl siloxane elastomer via replica casting using a LSFL-I-covered stainless steel master (Top). Elastic sample deformation with a resulting strain of (**a**) 0%, (**b**) 12%, (**c**) 24%, (**d**) 36%, (**e**) 48%, and (**f**) 60% results in a reversible mechano-responsive color change in the visible electromagnetic spectrum from blue to red (Bottom). In each individual photograph, the direction of the mechanical deformation (vertical) is perpendicular to the direction of the LIPSS-ridges (horizontal). Reprinted from [93], with permission from Elsevier.

One optical approach to how the structural colors of the grating-like LIPSS topography can be controlled via mechano-responsive changes was explored in [93] upon elastic deformation of LSFL-covered polymers or upon plastic deformation of LSFL-covered metals. Figure 7 exemplifies the changes of the structural colors of LIPSS (that were previously transferred via replica casting from a fs-laser-processed steel master to a polymeric elastomer sample) upon its linear mechanical deformation in the direction perpendicular to the LIPSS ridges. Here, a linear elastic deformation (strain) resulted in a linear increase (redshift) of the wavelengths of the structural color diffracted from the polymer surface covering the ranges from blue to red in the visible electromagnetic spectrum (see the bottom part of Figure 7). Such a change of structural colors of LIPSS on polymers or metals through elastic or even plastic mechanical deformations may be of particular interest for the development of strain gauges, novel switches and safety devices, or for the early detection of material failure [5,93].

In a distinct optical approach, the grating-like spatial characteristics of LSFL can be imprinted in the sub-ablation regime to a phase change material (PCM) such as GeTe [94] or Ge_2_Sb_2_Te_5_ [95], allowing the stripes/ridges of the LIPSS to be represented either by the amorphous or by the crystalline phase of the PCM. In this way, by proper choice of the laser irradiation parameters, micron-sized spots with an optically encoded LIPSS patterns can be written and erased again [95,96]. Such optical patterns may be used for information encoding or act as unique safety features.

Another possibility for control relies on a LIPSS-induced anisotropy of the electric properties, e.g., via a periodic modulation of the electric conductivity or resistivity on the sub-micrometer scale. Such effects were observed for LSFL in transparent conductive oxide layers through local changes of the stoichiometry [76], or they can be induced by local oxidation effects [53,56], or even by structural changes in semiconductors such as laser-induced amorphization [52,94], or by crystalline defects [97].

**Question** **8.**  **Which are the Most Suitable Methods to Visualize LIPSS Formation?**

Given the typical periods of LIPSS in the sub-micrometer to micrometer range, it is not possible to resolve these surface features by the naked eye. They modify, however, the properties of surfaces and can be visualized in manyfold ways.

***Ex-situ visualization:*** In most cases, LIPSS are visualized ex-situ, i.e., in a subsequent step after their processing. The most prominent and simple method is optical microscopy (OM). However, its spatial resolution is typically limited to ~*λ*/2 and, thus, it already requires a very good optical microscope operated at the optical diffraction limit to directly image LSFL. HSFL cannot be resolved by common OM. This limitation can be overcome by employing scanning electron microscopy (SEM), taking benefit of the reduced de Broglie wavelength of the electrons. SEM is capable of spatially resolving and also imaging the tiny HSFL. Nevertheless, OM and SEM typically lack the necessary depth-resolution to quantify the modulation depth of LIPSS. To obtain that information, atomic force microscopy (AFM) can be used by experienced operators to obtain a surface topography *z*(*x*,*y*). The most complete information on LIPSS can be provided by transmission electron microscopy (TEM) that, prior to the analysis, requires the preparation of a cross-sectional lamella cut through the LIPSS-profiles, typically obtained by focused ion beam (FIB) etching and subsequent milling of the extracted surface lamella to reach electron beam transparency. The FIB-based sample preparation in particular requires highly experienced operators and is very time consuming. However, once such a sample is successfully prepared, TEM can directly reveal its material structure either by high-resolution imaging or by selected area electron beam diffraction in order to visualize even amorphous or chemically altered surface layers, crystalline defects, or the grains of the material’s texture [63,97].

Regarding spatially and chemical resolved analyzes, some comments must be made. Most convenient and often available along with SEM is energy dispersive X-ray spectroscopy (EDX, also referred to as EDS) that is capable of providing spatial maps of (relative) elemental concentrations. While the spatial resolution is high enough to resolve both LSFL and HSFL, the information depth of EDX is usually in the few micrometer range (depending on the electron acceleration voltage used). Thus, significant X-ray signal contributions arise from the bulk material underneath the superficial LIPSS topography. Additionally, care must be taken with chemical quantifications (at%, mass%) since the underlying models usually assume perfectly flat surfaces without any topographic (e.g., LIPSS) corrugations. Hence, even if appealing, the use of EDX for the chemical characterization of LIPSS is not recommended here. Three surface analytical methods that exhibit the necessary surface sensitivity featuring information depths of <10 nm are X-ray photoelectron spectroscopy (XPS), Auger electron spectroscopy (AES) and glow-discharge optical emission spectroscopy (GD-OES). While standard XPS and GD-OES do not provide lateral resolution in the sub-µm range, AES provides high-resolution imaging capabilities and can be employed as scanning Auger microscopy (SAM). In combination with ion-depth profiling, the spatial resolution and the depth resolution are good enough to analyze LSFL and HSFL in detail [56]. Other surface characterization techniques such as micro-Raman spectroscopy (µ-RS) or X-ray diffraction (XRD) exhibit neither the necessary spatial nor the depth resolution to unfold individual LIPSS ridges but may complement other techniques. Table 1 provides a comparison of the suitability of different ex-situ LIPSS visualization techniques.

***In-situ visualization:*** There are, however, also in-situ approaches to study the dynamics of the formation of LIPSS. The potential to use light being diffracted at the grating-like surface topography of LIPSS to study the dynamics of their formation was already recognized during the early 1980s [98,99,100]. However, at that time, the available pulse durations in the ns-range limited the temporal resolution of these pump-probe diffraction experiments and phase transitions such as melting and ablation were temporally convoluted. This changed with the availability of ultrashort laser pulses that allowed the dynamics of LIPSS formation to be revealed on ultrashort timescales either in optical diffraction experiments [35,101], in transient optical scattering experiments [102,103], or even in time-resolved optical microscopy [52,104]. However, using probe wavelengths in the optical spectral range limits the detection of LIPSS to periods exceeding several hundreds of nanometers. This limit was overcome in 2010 by employing ultrashort (<30 fs) 13.5 nm wavelength coherent XUV probe radiation of a free electron laser (FEL) at the German electron synchrotron DESY in Hamburg [105]. The latter work still represents the ultimate approach to resolving LIPSS in space and time.

***In-line monitoring*:** For practical purposes and in industrial applications, however, such time-resolved in situ pump-probe experiments are by far too complex. Here it is sufficient to take benefit of the optical diffraction effect and implement an in-line monitoring of the diffracted signal with a photodetector (photodiode or video camera), e.g., by tracking the diffracted signal amplitude (photodiode) or the shape and position of the diffraction pattern (camera) pulse-by-pulse during LIPSS processing. For a good signal quality, many LIPSS ridges should ideally be illuminated in parallel by the probing radiation. In the pertinent literature, in-line monitoring of laser-processed periodic surface structures was recently reported by Michalek et al. [106] for LIPSS and by Teutoburg-Weiss, Schröder et al. [107,108] for DLIP structures.

**Question** **9.**  **What Factors are Currently Hindering a Transfer to Industry?**

Currently, LIPSS-based surface structuring primarily takes place on a laboratory scale. The main requirements for a successful transfer to industrial manufacturing processes and applications are the reliability, scalability, and efficiency of the structuring process.

From the technological perspective, LIPSS generation is characterized by a relatively simple experimental setup compared to alternative surface structuring processes. Structural sizes ranging from a few 10 nm up to several µm can be generated in a single-step direct-writing process without the need for cost-intensive vacuum technology, complex optical setups or chemicals. Nevertheless, a main disadvantage of LIPSS is given by the limited modulation depth of the structures (few 100 nm at maximum) that cannot be set independently of their spatial period. In addition, the regularity of ablative LIPSS depends on many parameters and, with the exception of some metals, is difficult to control (see Question 4). This is where alternative and more complex methods such as lithography and DLIP have advantages. In order to be able to produce LIPSS reliably and reproducibly for the numerous potential applications including optics, microfluidics, tribology, and medicine, much work is being done on a detailed understanding of the formation process and the exact correlation of all influencing parameters involved (material, process, laser irradiation). However, the theoretical modelling is very complex due to the diversity of the processes entailed, which is why the predictability—despite numerous model approaches (see Question 10) and the availability of powerful computing technology—is still insufficient for industrial applications. The practical optimization of LIPSS-based surface structuring, therefore, still takes place today, 56 years after the first observation of LIPSS by Birnbaum [3], mainly through an iterative trial and error procedure. However, once the process is established via identifying suitable laser processing parameters (wavelength, fluence, number of effective pulses per focal spot area, etc.), the LIPSS pattern can be in-situ or even in-line monitored via its diffraction signature on a photodetector (see Question 8).

In terms of reliability and durability, in addition to the structuring process, the resulting surface functional properties must also be considered for transfer to industry. In this context, it was shown, e.g., that some LIPSS-covered surfaces are subject to a certain ageing concerning their wettability [59]. For this reason, the desired surface functionality is not permanently available (see Question 6). There has been intensive research activity in this area in recent years, with increasing focus on the influence of the surface chemistry both during the formation process and during the subsequent sample storage (see Question 5).

The above-mentioned scalability of the structuring process to industrial requirements is closely linked to the process efficiency. The main objective of producing functional surfaces of industrially relevant areas (m^2^-range) cost- and time-efficiently requires the further development of laser radiation sources as a central element. The major advantage here is the relatively low pulse energy required for LIPSS formation, which is why the focus of future developments of laser systems from the LIPSS perspective must rely on high repetition rates. Since LIPSS formation as a multi-pulse process generally requires a specific effective number of pulses hitting the material surface, an increase in the pulse repetition rate automatically leads to a shortening of the process times, i.e., an increase in the surface area that can be structured in a given time unit.

Regarding the rapid progress in laser technology, it was noted recently by Han et al. [109] that the average power of ultrashort lasers obeys an analog to the famous Moore’s Law that framed the developments in the field of computer technology for decades. Since the year 2000, a doubling of the average output power of ultrashort (sub-ps) pulsed lasers was observed every two years, i.e., a growth rate ~2^Q/2^ with *Q* being the number of the years from beginning of the trend [110].

**Figure 8:**
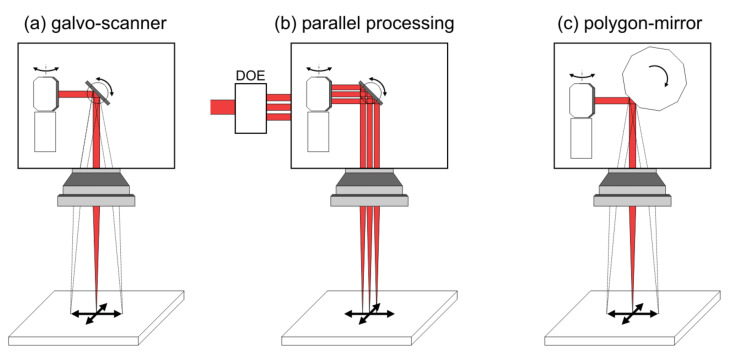
Enhancement of process efficiency: (**a**) galvanometer-scanner processing, (**b**) parallel processing by using a galvanometer-scanner and multiple laser beams generated by a diffractive optical element (DOE) and (**c**) ultra-fast processing with polygon-mirror-based scanner systems.

Currently, employing galvanometer-scanner technology (Figure 8a), the maximum areal process rates are in the order of m^2^/min [111] and could be increased to m^2^/s when using GHz laser pulse repetition frequencies along with fast and smart beam management strategies. However, this new milestone in process efficiency demands suitable, flexible, and precise scanning technologies with scanning speeds of around 100–1000 m/s to be able to distribute the pulses over the material surface. Such requirements are currently only met by polygon-mirror-based scanner systems [111,112]; see Figure 8c. Other approaches are based on spatial beam shaping in order to increase the simultaneously processed area, for example, by means of a line focus or via parallel processing by splitting the incoming laser beam into several processing beams (Figure 8b).

However, in all these high-throughput approaches it must be carefully checked in the future whether the residual heat-load imposed on the processed sample upon high-speed laser-processing of LIPSS remains small enough to avoid heat-accumulation effects.

**Question** **10.**  **Quo vadis LIPSS Modelling?**

The fundamental problem of modelling LIPSS arises from its extreme intrinsic multi-scale nature that includes time scales covering a few femtoseconds and sometimes up to microseconds [seven orders of magnitude] along with spatial scales ranging from atomic dimensions (~0.1 nm) up to extended patterns (sometimes processed on m^2^-sized areas) consisting of individual (quasi)periodic building blocks featuring “unit cell” sizes (LIPSS periods) typically between ~100 nm and 10 µm [five orders of magnitude] in three spatial dimensions (*x*, *y*, *z*). Apart from these dimensional challenges, a chain of complex physical processes is involved and must be rendered in temporal and spatial constraints.

As a first step, the electronic system of the solid is excited by the laser radiation incident to the rough surface of a sample-specific topography. Apart from scattering and near-field enhancement effects, in case of semiconductors and dielectrics nonlinear excitation of electrons in the conduction band (CB) may take place if intense ultrashort laser pulses are employed. These CB carriers can locally and transiently (intra-pulse) change the dielectric permittivity of the laser-excited material (ε*). If certain conditions are fulfilled, so-called surface electromagnetic waves may be excited at the surface, e.g., through collective electron excitation modes such as Surface Plasmon Polaritons (SPP), acting back with the laser radiation and creating localized currents and re-irradiating electromagnetic fields [113,114].

Once excited into a non-equilibrium state in the CB, the electrons start to diffuse spatially while transferring their excess energy to the lattice of the solid via electron–phonon relaxation. The latter occurs within characteristic times *τ*_e-ph_ between a few tenth and tens of picoseconds, depending on the specific material. Once the lattice is heated, additional thermal processes can occur, including phase transitions such as melting and evaporation, heat flows, the propagation of stress- or pressure-related waves such as rarefaction- or shock-waves, spallative material removal, disintegration of the laser-excited material into clusters, nano- and microparticles, hydrodynamic motion of the residual melt layer driven by thermo- or chemocapillary forces, defect formation, or re-solidification.

**Figure 9:**
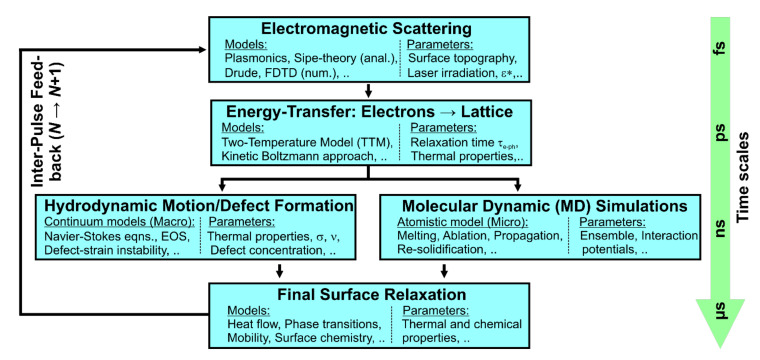
Scheme of the current state of analytical and numerical modelling of LIPSS.

Figure 9 orders in a flow chart the present state of theoretical modelling of LIPSS. For more details on specific models, the reader is referred to the recent review article [10] and the references therein. In brief, the electromagnetic excitation and scattering at early times (fs) were successfully modelled by J.E. Sipe and co-worker during the 1980s while developing a rigorous analytical theory of the absorption of optical radiation by a rough surface [33]. Later this approach was extended by combining it with a Drude model in order to address intra-pulse transient changes of ε* of the laser-excited material, the so-called Sipe–Drude model [39,115]. The Sipe-theory is restricted to the near-surface region, i.e., to a “selvedge” extending about *λ* in depth. The theory does not include any inter-pulse feedback processes that are usually involved in the formation of LIPSS [116]. The restriction to near-surface regions can be overcome numerically by employing Finite-Difference Time-Domain (FDTD) calculation to solve Maxwell’s equations for materials and surfaces of almost arbitrary topography [34,117].

The transfer of energy from the laser-excited electronic system to the lattice is typically modelled via a Two-Temperature Model (TTM), treating the temperatures of the electrons and the lattice individually but in a coupled way [118]. In an alternative and more rigorous approach, the energy transfer can be treated by a kinetic Boltzmann approach that takes properly into account the electronic band structure of the material and possible electron emission, and thermalization processes [119,120]. Once spatial temperature fields are established for the materials phases involved (liquid, solid), in a macroscopic continuum approach the Navier–Stokes Equation (NSE) may be employed in a compressible [36] or incompressible [121] form for calculating numerically material displacement in the laser-induced melt pool. Considering the continuity equation and the specific boundary conditions of these partial differential equations, this can include thermocapillary effects, such as the Marangoni– or Rayleigh–Taylor instabilities, via the additional equation of states (EOS) the propagation of pressure, shock, rarefaction and capillary waves, surface tension effects, etc. Alternatively, the material response can be treated on an atomistic (microscopic) scale via Molecular Dynamic (MD) simulations [122,123,124]. After re-solidification and subsequent chemical effects, such as surface oxidation, the sample surface finally reaches a relaxed state that can be used again as an input to a new cycle of simulations for propagating the impact of the *N*^th^ to the (*N* + 1)^th^ laser pulse, i.e., inter-pulse feedback can be implemented in a feedback loop.

Remarkable efforts were made in the modelling of LIPSS, particularly during the last decade. While the pioneering works of Sipe and co-workers were widely restricted to the deposition of energy to the material, the plethora of “thermal material response pathways” was not explicitly included. This has changed in the last decade, where the FDTD simulations in particular have turned out to be useful when combined with additional material-response models. In this way, state-of-the-art LIPSS modelling is at present based on combined FDTD-TTM-NSE [36] or TTM-MD [122,123,124] models. Additionally, remarkable efforts regarding an explicit understanding of how exactly SPP couple to a corrugated surface were recently presented [125,126,127].

## 3. Gleanings towards the Bright Future of LIPSS

At the end of the 1980s, the topic LIPSS appeared to be “dead” again: rigorous theories on LIPSS formation had been developed while relevant applications had not been found at this time. This paradigm changed at the latest at the turn of the millennium with the discovery of the sub-diffraction limit HSFL. Starting from that seed, the academic and industrial interest in LIPSS intensified again, particularly since nanotechnology was one of the most relevant topics at that time and ultrashort lasers became widely commercially available in research laboratories. Two different “schools” re-animated the LIPSS topic during the following two decades and tried to associate their origin either with (i) electromagnetic effects seeding the characteristics of LIPSS already during the ultrashort laser pulse durations, or with (ii) matter re-organization effects driving self-organization after the laser irradiation process. Jürgen Reif, to whom this Special Issue is devoted, very actively promoted research in the direction of self-organization and made important contributions here [128,129,130,131]. Over the last few years and with the development and numerical implementation of advanced multi-scale models involving various physical effects, the two LIPSS schools are merging [10,11] and new applications of LIPSS are currently systematically being screened and explored [5,6].

## Figures and Tables

**Table 1:** Comparison of suitability of different surface characterization techniques (for their abbreviations refer to the main text) for the visualization of LIPSS. Symbols/abbreviations: ✓: yes, ✕: no; l: low; m: medium; h: high.

	Characterization Technique									
	OM	SEM	AFM	TEM + FIB	EDX	SAM + ion	ToF-SIMS + ion	XPS + ion	GD-OES	µ-RS
Lateral resolution for LSFL?	✓	✓	✓	✓	✓	✓	✓	✕	✕	(✓)
Depth resolution for LSFL?	✕	✕	✓	✓	✕	✓	✓	✓	✓	✕
Lateral resolution for HSFL?	✕	✓	✓	✓	✓	✓	✓	✕	✕	✕
Depth resolution for HSFL?	✕	✕	✓	✓	✕	✓	✓	✓	(✓)	✕
Large area inspection possible?	✓	✓	✕	✕	✓	✓	✓	✓	✕	✕
Time consumption/resources	l	m	m	h	m	h	h	m/h	l	l

## Data Availability

Data sharing not applicable as no original datasets were generated or analyzed in the current article.
